# Inflammatory Markers and Intimal Media Thickness in Diabetics with Negative Myocardial Perfusion Scan

**DOI:** 10.4021/jocmr2009.05.1239

**Published:** 2009-06-21

**Authors:** Douraid K. Shakir, Ibrahim Mohmmed, Mahmood Zarie, Dawod Al Kateeb, Abdul Salim Kiliyanni, Jassim Al Suwaidi

**Affiliations:** a Department of Cardiology and Cardiovascular surgery, Hamad Medical Corporation, Qatar.; b Department of Endocrinology, Hamad Medical Corporation, Qatar.; c Department of Radiology, Hamad Medical Corporation, Qatar.

## Abstract

**Background:**

We compared the type and duration of diabetes mellitus (DM), patient demography, high sensitivity C-reactive protein (hsCRP), Homocysteine and other variables with IMT, to determine if these markers were correlated in diabetes (in whom technetium myocardial perfusion scan were negative) and would it be appropriate biomarkers for arthrosclerosis detection in this group of diabetics.

**Methods:**

Forty patients with DM, without CHD history, were screened with stress sintigraphy imaging using 2 days stress/rest Technetium 99 tetrafosmin protocol, employing the standard Bruce protocol. Echocardiography study requested for each patient, two blood samples for hsCRP, were requested for each candidate three weeks apart, Lipid profiles, plasma homocysteine, and hemoglobin A1C were also requested. Finally Intima-media thickness were measured for all patients.

**Results:**

There were no relationships between hsCRP level and DM duration or with the type of DM; also there were no relation between DM duration and homocysteine or between DM type and Homocysteine. Intimal media thickness was increased proportionally with the serum level of Homocysteine.

**Conclusions:**

This study did not show any role for the inflammatory markers in predicating the presence of coronary artery disease in participants with DM, without medium size artery disease, which may support that DM is not the only player in initiating atherosclerosis.

**Keywords:**

Diabetes mellitus; Inflammatory markers; C-reactive protein; Myocardial ischemia; Homocysteine; Intima-media thickness

## Introduction

Cardiovascular disease is the leading cause of mortality in diabetic patients, accounting as many as 80% of diabetic patient deaths [1]. The risk of myocardial infarction (MI) in diabetic patients is similar non-diabetic patients with a history of a previous MI [2]. Post-mortem studies on diabetics suggest a high prevalence of coronary atherosclerosis, even among those without clinical coronary heart disease (CHD). Advanced coronary lesions are found in about 75% of diabetic patients without clinically apparent CHD and > 50% of asymptomatic individuals also had multi-vessel disease [3]. Moreover, the prevalence of silent ischemia among asymptomatic diabetics is high, ranging from 20 to > 50% [4]. The incidence of Diabetes Mellitus (DM) among Qatari patients with acute myocardial infarction is 54% [5].

Biomarkers are used for risk assessment stratification for patient. These include increased high-sensitivity C-reactive protein (hsCRP), an inflammation marker; homocysteine, indicative of endothelial function and oxidative stress; and urinary albumin : creatinine ratio, an indicator of glomerular endothelial function [6]. Increased intima-media thickness (IMT) of the common carotid artery, measured by high resolution B-mode ultrasound imaging, occurs early in the atherosclerosis process and generally precedes plaque development and stenosis in the arterial wall as a marker for the presence of atherosclerosis. Also, IMT is associated with a higher prevalence of symptomatic and asymptomatic CHD and correlates with future development of myocardial infarction and stoke [7]. We compared the type and duration of diabetes mellitus (DM), patient demography, HsCRP, Homocysteine and other variables with IMT to determine if these markers were correlated in diabetes; would it be appropriate biomarkers for early arthrosclerosis detection in diabetics and lastly their behavior and inter- relationship of these markers with diabetes mellitus disease process.

## Materials and Methods

Consecutive forty patients with DM, with no history of ischemic heart disease were referred from endocrinology clinics and participated in the study from January 2003 till November 2006. A written consent was obtained by all participants. Medical history, physical examination, body weight, height, and vital signs were all recorded. Electrocardiogram and Chest X-ray were performed for all patients. Patients were screened with stress sintigraphy imaging using 2 days stress/rest Technetium 99 tetrafosmin protocol, employing the standard Bruce protocol. The Single photon emission computed tomography (SPECT) images were acquired using a dual-head gamma camera (E. cam, Siemens) to exclude CHD and medium size artery disease. Echocardiography was performed for patients using Acuson sequoia C512, to assess the average ejection fraction (EF), measured by 2 and 4 chambers and apical views. Two blood samples for hsCRP, after excluding aberrant infections by history, were requested for each candidate three weeks apart and were analyzed by IMMAGE kite, Immunochemistry system (Beckman Coulter). Lipid profiles, plasma homocysteine, and hemoglobin A1C were also requested. Intima-media thickness was measured for each patient by High resolution B mode, colour Doppler, and pulse Doppler Ultrasonography of both carotid arteries were performed with an ultrasound machine (Logiq 9, General Electric Company) equipped with a 10 MHz linear array transducer. Patients were examined in the supine position with the head tilted backwards. After the carotid arteries were located by transverse scans the probe was rotated 90 to obtain and record a longitudinal image of the anterior and posterior walls. The maximum IMT was measured at the far wall of the both common carotid arteries during end diastole, 2 cm proximal to the bifurcation, from a longitudinal scan plane that showed the intima-media boundaries most clearly. The longitudinal view of the normal carotid wall demonstrates two nearly parallel echogenic lines. The inner line represents the lumen-intima interface and the outer line represents the media adventitia interface. The distance between these lines is the combined thickness of intima-media complex (I-M complex). IMT may be the most sensitive marker for the earliest stages of atherosclerosis and it is considered to be a marker of generalized atherosclerosis.

In healthy adults, IMT ranges from 0.25 to 1.5 mm [8], and values > 1.0 mm are often regarded as abnormal [9]. IMT has been proposed as a quantitative index of atherosclerosis of value in monitoring disease progression and the effects of treatment and as a surrogate end point in clinical trials. Average measurements of left and right carotid arteries were used. The study protocol was reviewed and approved by the institutional review board of the Hamad Medical Corporation. All tests and procedures were free of charge for participants. The data were handled by the biostatistics department, Hamad Medical corporation using SPSS software, the P value were calculate by Chi-square, T test, Correlation Coefficient and linear regression with Confidence intervals of 95%.

## Results

### Participant demographics

Forty patients were enrolled in the study. Two patients had positive results with stress technetium myocardial perfusion scan were excluded from the study; while thirty eight patients participated in the study had negative results for myocardial radio-isotope scan were included in the study, 17 females and 21 males, with mean age of 54 years (41-87 years of age). Fifteen cases were DM type one while the rest were DM type two. DM duration ranged from 2 to 30 years, with an average of 11.3 years. Twenty six cases were hypertensive ranging from 1 to 15 years, with an average of 6.5 years. Body mass index (BMI) ranged from 22.7 - 48.59, with an average of 30.67. Three cases had a normal BMI, while fifteen cases were overweight with BMI > 25, and remaining were obese with BMI > 30. Out of 40 patients, 32 patients never smoked, six were current smokers, and only two patients were ex-smokers. Ejection fraction ranged from 50 to 72%, average of 63.2% (Table 1). None of the patients showed wall motion abnormalities.

**Table 1:** Baseline characteristics of study participants

Characteristics	Data	
Sex distribution	Male	21
	Female	17
Age	Range	41-87 years
	Average	54 years
Technetium study	Positive	2
	Negative	38
Ejection fraction	Range	50 - 72%
	Average	0.632
Type of DM	Type 1	13
	Type 2	25
Duration of DM	Range	2 - 30 years
	Average	11.3 years
Hypertension cases	with	26
	without	12
Duration of Hypertension	Range	1 - 15 years
	Average	6.3 years
Smoking	None	30
	Present or Ex	8
BMI	Range	22.7 - 48.59
	Average	30.677
hsCRP	Range	0.327-6.3 mg/dl
	Average	0.9777 mg/dl
Homocysteine	Range	3 - 93 μmol/l
	Average	12.217 μmol/l
IMT	Range	0.45 - 1.8 mm
	Average	0.877 mm
HbA1c	Range	5.1 - 11.2 %
	Average	0.07637

### High sensitivity C-reactive protein (hsCRP) analysis

The two cases with positive stress sintigraphy imaging for ischemic heart disease had HsCRP level of 0.274 and 0.417 mg/dl respectively both values were below the reference value of < 0.744 mg/dl (P = 0.35).

**Figure 1:**
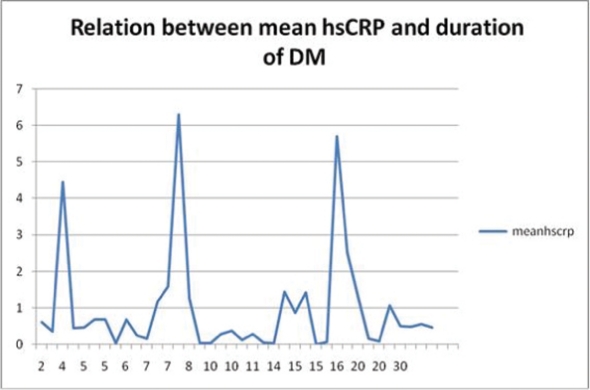
Relationship between mean hsCRP and DM duration. Axis X represents duration of DM in years, axis Y represents level of hsCRP in mg/dl.

More than half of the cases (26 patients) showed high sensitivity C-reactive protein levels (hsCRP) below reference values and only 12 cases had hsCRP levels more than 0.744 mg/dl. HsCRP levels were ranging from 0.327 to 6.3 mg/dl, with an average of 0.9777 mg/dl. There was no relationship between hsCRP level and DM duration the Pearson Correlation co-efficient equal to 0.204 and P value of 0.225 (Fig. 1) or with the type of DM (P = 0.37) (Table 2). Also, no relationship was found between hsCRP and BMI with Pearson Correlation co-efficient of 0.112 and P value of 0.527. Finally no relation was found between CRP and smoking in this present study, (5 smoker and the rest were none smokers), P = 0.37.

**Table 2:** Statistical relationship between Diabetes mellitus duration and type with different inflammatory markers and arterial Intimal media thickness

Inflammatory markes	DM duration (P value)		DM type (P value)	
HsCRP	0.225	NS	0.37	NS
Homocysteine	0.92	NS	0.53	NS
IMT	0.971	NS	0.49	NS

NS for not significant

### Homocysteine analysis

Homocysteine levels were measured for all patients and ranged from 3 to 93 μm/L, average was 12.217 μm/L. Only five cases were higher than the reference value of 20.44 μm/L. The two cases with positive stress sintigraphy imaging for ischemic heart disease had Homocysteine level of 7 and 11 μm/L respectively which is below the reference value of 20.44 μm/L (P = 0.058).

**Figure 2:**
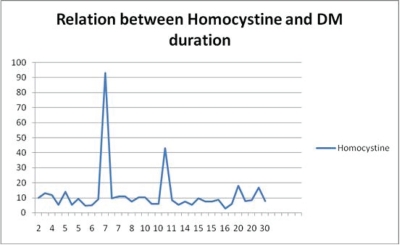
Relationship between homocysteine and DM duration. Aixs X represents the duration of DM in years, axis Y represents the Homocystine level in μmol/l.

There was no relation between DM duration and homocysteine (P = 0.92; Fig. 2) or between DM type and homocysteine (P = 0.53) (Table 2). Also, there were no relations between CRP and homocysteine levels in this sample with Pearson Correlation Co-efficient of 0.64 and P value of 0.708.

### Intimal media thickness (IMT) analysis

Intimal media thickness (IMT) was measured for all cases by the same operator. Values > 1.0 mm are regarded as abnormal [9]. IMT values ranged from 0.45 mm to 1.8 mm, average of 0.88 mm. Sixteen cases had an IMT of equal to or less than 1mm, while the rest (22 cases) were more than 1 mm. Neither DM duration nor the DM type related to IMT (P = 0.971, P = 0.49, respectively) (Table 2). Only one case with positive stress sintigraphy imaging had the IMT measured with avalue of 1.1mm making the P = 0.39.

**Figure 3:**
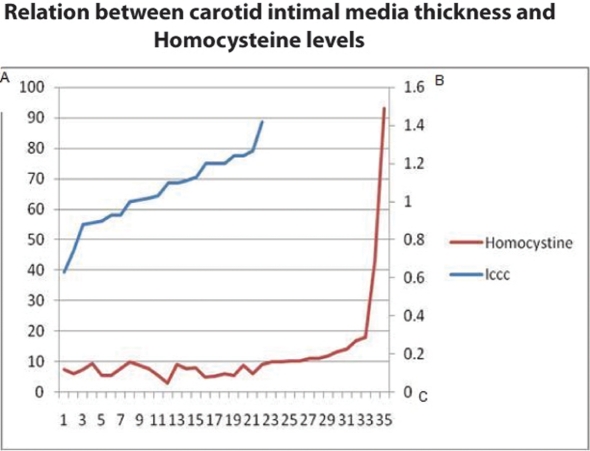
Relation between carotid intimal media thickness and Homocysteine levels. Axis A represent Homocystine level in μmol/l, axis B represent Intimal Media thickness in mm, axis C represent the cases.

Intimal media thickness was increased proportionally with the serum level of homocysteine with Pearson correlation co-efficient of 0.414 and P value of 0.050 (Fig. 3).

### Hemoglobin A1c analysis

The Hemoglobin A1c, ranged from 5.1 to 11.2 %, with an average of 7.6%. Out of the thirty-eight cases with no IHD, only two cases had HbA1c less than 6%. HbA1c levels suggest that most of our patients were not effectively controlling their blood glucose levels. This should theoretically have an impact on CRP; but we did not detect any significant differences. HbA1c was used as a parameter for overall blood glucose control. Regarding the two cases with positive stress sintigraphy imaging, one had HbA1c level of 5.8 while the other got a level of 9.1%.

## Discussion

Diabetes Mellitus leads to excess free fatty acids, hyperglycemia, and insulin resistance together these increase oxidative stress, protein kinase C activation, and receptors for advanced glycation end product activation. The total sum of these effects results endothelium dysfunction, mediated through many pro-inflammatory factors, causing inflammation and atherosclerosis [10]. This study examined inflammatory markers and IMT in DM patients without medium size artery disease, to determine if these markers were correlated with diabetes and would be appropriate biomarkers for early arthrosclerosis detection and whether there is inter-relationship between these factors and their behavior with the disease process.

Two cases were positive for IHD confirmed by radio-isotope myocardial scan and in both cases the results for inflammatory markers were low; but due to small sample, a solid conclusion regarding the relation between the level of inflammatory markers and presence of CAD should not be made.

C-reactive protein (CRP) is an acute-phase reactant produced by hepatocytes in response to a wide range of stimuli. Circulating at low concentrations in healthy individuals, CRP rises dramatically in response to infection, inflammation, and injury. CRP is used in clinical settings as part of the diagnostic workup to monitor disease status and treatment results. Studies suggest elevated CRP levels may predictive for cardiovascular disease and diabetes [11, 12]. In agreement with our findings, Haverkate et al [13] from the Angina Pectoris Study group, found no association between CRP and diabetes. The lack of association maybe due to increased BMI in DM and the presence of acute and chronic infections like diabetic foot [14-16].

Although previous studies showed CRP was correlated with BMI [13, 17], the magnitude of the association was not always presented. The European Concerted Action on Thrombosis and Disabilities (ECAT) Angina Pectoris Study found that CRP and BMI were significantly associated among 2,121 patients with angina pectoris, but no detailed analyses were presented [13]. The reasons for the apparent association between CRP and BMI are not clear. In the present study, we did not find a relationship between DM duration (without CHD) and BMI or a relationship between CRP and BMI.

Why other studies found an association between DM, BMI and CRP could be due to several factors. First, individuals with obesity are at increased risk for various chronic diseases, several are also characterized by elevated CRP. Second, subclinical disease may be responsible for the observed association. Third, obesity could be accompanied by an inflammatory component unrelated to accompanying clinical or subclinical pathology.

In the Cardiovascular Health Study, CRP was significantly associated with diabetes in nearly 190 subjects who never smoked, but not in nearly 180 subjects who had smoked [18]. In the present study, such relationship was not found likely due to the small participant number and few participants were smokers as others have same findings [13], this brings the need for more intensive research regarding these negative results with larger groups of patients.

Clinical evaluation for homocysteine may be an indicator for atherosclerosis when combined with a family history of atherothrombosis, in the absence of other major risk factors. Homocysteine evaluation is also likely to more prevalent among patients with renal failure [19]. Patients with severe hyperhomocysteinemia may have a variety of symptoms including a high incidence of vascular pathology that may result in early death from myocardial infarction, stroke, or pulmonary embolism. Biochemical and pathological studies in homocystinuric children concluded that elevated blood homocysteine may cause early arteriosclerosis. Observations in nearly 80 clinical and epidemiological studies suggest elevated homocysteine is a risk factor for atherosclerotic vascular disease and for arterial and venous thromboembolism [19]. In the present study, there was neither any relation between DM type and duration with homocysteine nor any relation between CRP and Homocysteine; yet we have some cases had DM for more than 30 years. These findings could be explained on a genetic variation and predisposition as stated below.

Increased intima-media thickness (IMT) of the carotid artery, as measured by high-resolution B-mode ultrasound imaging, occurs early in the atherosclerotic process and precedes the development of plaque and stenosis in the arterial wall [20, 21]. Hence associated with a higher prevalence of symptomatic and asymptomatic CHD and predisposes patients to myocardial infarction or stroke [22].

Diabetic patients exhibit a higher degree of early atherosclerosis than normal glucose-tolerant subjects, matched for age and sex. Data suggests hyperglycemia, together with other risk factors (particularly dyslipidemia), may cause intimal-medial thickening in the early phases of diabetes [23]. Yet we did not find a relationship between the duration or type of DM with IMT. However, DM’s involvement in atherosclerosis is complex and involves genetic predispositions, as well as, environmental influences [7]. The level of Homocysteine increased proportionally in this study with the IMT and this finding is consistent with many other studies that document the relation between these parameters and its relation to DM and IHD and the effect of such combination on atherosclerosis progression, thrombo-embolism and total cardiovascular morbidity and mortality [24-27].

The ratio of total cholesterol to high density lipoprotein is regarded as one of the strongest independent predictors for developing peripheral arterial disease [28]. In this present study, we found no relationship between the TC/HDL-C with duration and DM type and no correlation between IMT, BMI, and CRP with TC/HDL-C. These findings are reasonable as study participants did not exhibit medium size arterial disease. Also, this study did not show any role of inflammatory markers in participants with DM, without medium size artery disease, which may support that inflammation in DM is not the solo player in initiating atherosclerosis but other factors may have a role in accelerating the atherosclerosis process as other studies had suggested [7]. This finding is not accepted by many endocrinologist or cardiologists; hence we need more work concentrating on these negative results to clear up the clouds. As negative results will not initiate more research as positive results which sometimes taken as granted; but in this critical area of high risk patients one ought to be more accurate in predicting and even treating cases not on solid grounds.

The small sample of the study due to difficulties in logistics and patients acceptance to participate in such kind of studies limits the value and power of the results; yet it may contribute to the flow of the studies made in the same line and add some data to the global effort for early detection and monitor the progression of atherosclerosis. We combined the two types of DM in the study as we did not look for statin therapy in our group of patients together as we had small sample that dividing this sample will make the results more biased.
